# MnTEdb, a collective resource for mulberry transposable elements

**DOI:** 10.1093/database/bav004

**Published:** 2015-02-27

**Authors:** Bi Ma, Tian Li, Zhonghuai Xiang, Ningjia He

**Affiliations:** State Key Laboratory of Silkworm Genome Biology, Southwest University, Chongqing 400715, China

## Abstract

Mulberry has been used as an economically important food crop for the domesticated silkworm for thousands of years, resulting in one of the oldest and well-known plant-herbivore interactions. The genome of *Morus notabilis* has now been sequenced and there is an opportunity to mine the transposable element (TE) data. To better understand the roles of TEs in structural, functional and evolutionary dynamics of the mulberry genome, a specific, comprehensive and user-friendly web-based database, MnTEdb, was constructed. It was built based on a detailed and accurate identification of all TEs in mulberry. A total of 5925 TEs belonging to 13 superfamilies and 1062 families were deposited in this database. MnTEdb enables users to search, browse and download the mulberry TE sequences. Meanwhile, data mining tools, including BLAST, GetORF, HMMER, Sequence Extractor and JBrowse were also integrated into MnTEdb. MnTEdb will assist researchers to efficiently take advantage of our newly annotated TEs, which facilitate their studies in the origin, amplification and evolution of TEs, as well as the comparative analysis among the different species.

**Database URL:** http://morus.swu.edu.cn/mntedb/

## Introduction

Transposable elements (TEs) are mobile genomic DNA sequences that are ubiquitous in all living organisms (1). TEs were first discovered in the late 1940s (2), and were found to be a significant part of host genomes. TEs occupy large proportions of host genomes in organisms such as humans (44%) (3), flies (10–15%) (4), mice (40%) (5), wheat (80%) (6) and maize (85%) (7). TEs make up a large proportion of the C-value of the eukaryotic cell.

TEs represent one of several types of repetitive sequences and they can be classified into either of two classes according to the presence or absence of RNA as a transposition intermediate, retrotransposons or DNA transposons, respectively (8). Based on structural features, these classes can be further subdivided into orders, superfamilies and then families. Retrotransposons (class I) insert a copy of themselves into another location of the genome by a ‘copy and paste’ mechanism, using their encoded transcripts as an intermediate. Retrotransposons are commonly grouped into two distinct orders, LTR retrotransposons (Long terminal repeat retrotransposons: *Ty1/Copia*, *Ty3/Gypsy*, *Bel/Pao*, *Dirs*) and non-LTR retrotransposons (*LINE*, *SINE*) (8). DNA transposons (class II) use a ‘cut and paste’ mechanism and do not involve RNA as an intermediate. DNA transposons are commonly grouped into two main orders, terminal inverted repeats (TIRs) and *Helitrons* (9).

More and more evidence has unambiguously shown that TEs have a major impact on structural, functional and evolutionary dynamics of genomes (10–12). Meanwhile, the high degree of similarity and duplication of TE sequences presents difficulties in genome sequencing, annotation and analysis. Therefore, the identification of TEs will inform research into the influence of TEs on the structural, functional and evolutionary dynamics of the sequenced genomes.

*Morus* (mulberry) is the type genus of the cosmopolitan family Morceae (order Rosales), and has been used as an economically important food crop for the domesticated silkworm for a long time. However, little TE information for mulberry can be obtained from the public database. The draft genome sequences of *Morus notabilis* C.K. Schneid were available in 2013 (13), which provided the opportunity for identification of TEs in detail. In this study, TEs in mulberry were identified using comprehensive methods. All identified TEs were deposited in the developed database, MnTEdb. Some tools were also integrated for the analysis of TEs. MnTEdb can be used not only to study the origin, amplification and evolutionary dynamics of TEs in mulberry, but also for comparative analysis among different species to decipher the roles of TEs on genes and genomes.

## Construction and content of the database

### System implementation

MnTEdb was constructed using LAMP (Linux Ubuntu Sever 12.04, Apache 2, MySQL Server 5.5 and Perl 5.16.3/PHP 5.3), which is comprised of open source software and is one of the fastest ways to develop an enterprise-level database. All TE data and information were stored in MySQL tables and therefore response time is quick. The CGI (Common Gateway Interface) programs were mainly developed using Perl, JavaScript and PHP programming languages. The JBrowse Genome Browser, a fast, embeddable genome browser built with HTML5 and JavaScript, was used for manipulation and for display of positional relationships between genes and TEs in the MnTEdb database (14).

### Data sources

The new assembly of the mulberry genome was downloaded from the *Morus* genome database http://morus.swu.edu.cn/morusdb/. The Repbase Update collection (update 20130422) was downloaded from http://www.girinst.org/repbase/index.html (15). The *Viridiplantae* TE database was downloaded and retrieved from this Repbase. The Plant Repeat Database, including *Brassicaceae*, *Fabaceae*, *Gramineae*, and *Solanaceae*, was downloaded from http://plantrepeats.plantbiology.msu.edu/index.html (16). The RepeatPeps database of TEs was obtained from the RepeatMasker (http://www.repeatmasker.org update 20130422).

### Identification of putative TEs within the mulberry whole genome shotgun (WGS) assembly

An unmasked WGS assembly of mulberry was used as the input data source for TE detection (13). TE libraries of mulberry were generated using three approaches.

### *De novo* identification of TEs

*De novo* identification of TEs was performed using PILER (17) and RepeatModeler (http://www.repeatmasker.org/RepeatModeler.html, version 1.0.7). PILER-DF (17) analysis on the full assembly genome of mulberry was compared with itself using the PALS (http://drive5.com/pals/) algorithm with the default parameters. Because the genome is too large to align the entire sequence to itself, the genome was split into chunks small enough for PALS. Each chunk was aligned to itself. Then each different pair of chunks was aligned to each other. Families of the dispersed family were searched by using a minimum family size of three members and a maximum length difference of 5% between every two members in one family. The consensus sequence for each family was created after aligning the identified sequences with MUSCLE (version 3.7) (18). RepeatModeler assisted in automating the runs of RECON (19) and RepeatScout (20) to analyse the mulberry genomic database and used the output to build, refine, and classify consensus models of putative interspersed repeats. Repeats identified by RepeatModeler were filtered for low complexity using Tandem Repeats Finder (version 4.07b) with the default parameters (21).

### Signature-based identification of TEs

For LTR retrotransposons, LTR_STRUC (22) and LTR_FINDER (23) were used to search the new assembly of the mulberry genome with default parameters. For LTR_FINDER (23), the option –w 2 was used to get a table format output, which could be parsed to get the sequences based on the information of the elements. These *ab-initio* programs identify putative LTR retrotransposons based on diagnostic signatures, including LTRs and TSDs (Target Site Duplications). To be able to predict the location of the PBS (Primer Binding Sites), we constructed a database of tRNAs using tRNAscan-SE (version 1.3.1) (24). Default parameters for scanning eukaryotic genomes were used to predict the location of PBS using LTR_FINDER (23). Non-LTR LTR retrotransposons were identified by the pHMM based MGEScan-nonLTR program with default parameters (25). HelitronScanner (26) with default parameters was used for detecting *Helitron*. HelitronScanner was developed based on the LCV (local combinational variable) algorithm (27) and is considerably superior to previous *Helitrion* identification programs. LCVs are extracted from a training set of published *Helitrons*, and then the program scans the whole genome and scores how well the LCV matches. The putative *Helitrons* termini were determined based on matching scores. Consensus sequences of small non-autonomous DNA transposon elements were generated by using MITE-Hunter (28) with default parameters. The output files of MITE-Hunter included consensus TE sequences grouped into families. TIR and TSD structures of these sequences were manually checked using the MSA (multiple sequence alignment) files generated by MITE-Hunter.

### Similarity-based identification of TEs

The consensus sequences of the conserved DDE/D transposase domains of each DNA transposon superfamily were obtained from Dr Yaowu Yuan (29). These sequences were used as a query to search the mulberry genome using TBLASTN. The process was performed using the TARGeT pipeline (30), with an E-value cutoff of 0.01. Flanking DNA sequences within 10 kb upstream and downstream of the matched regions were retrieved. To determine the boundary of the full length of the putative elements, two closely related elements with their 20 kb flanking sequences were aligned using NCBI-BLAST 2 SEQUENCES (31). Usually, the boundary of a full-length element can be refined by identifying TIRs and TSDs around the breakpoint of a pair-wise alignment. Meanwhile, our own Perl scripts were also used to identify TIRs and TSDs based on features described by Yuan and Wessler (29). If the TIRs and TSDs of a putative element could not be determined, 1 kb of DNA sequences flanking the TARGeT matched region were retrieved to serve as a representative of the element.

### Definition of superfamily of putative TEs

The putative TE sequences output generated by all of the above approaches were used to create a unified custom repeat library that could be compared with previously characterized elements. All these repeats in the custom library were compared to a *Viridiplantae* TE database retrieved from Repbase (http://www.girinst.org/repbase/index.html, update 20130422) (15) and a Plant Repeat Database (http://plantrepeats.plantbiology.msu.edu/index.html) (16), using tBLASTx and BLASTn. The custom library was also compared to the RepeatPeps database of TEs that comes with RepeatMasker (http://www.repeatmasker.org, update 20130422) using BLASTx. If the E values of one repeat in the custom library showed at least 1.0e-5 with a common subject in at least two of the three databases mentioned earlier, the repeats were classified in a TE superfamily (32).

LTR retrotransposons typically contain open reading frames (ORFs) for GAG (a structural protein for virus-like particles) and POL (aspartic proteinase, reverse transcriptase, RNase H, and DDE integrase). The difference between the *Gypsy* and *Copia* superfamily is the order of RT and INT in the POL (8). The EMBOSS Getorf program was used to obtain the ORFs of the putative LTR retrotransposons (33). All the candidate LTR retrotransposons were searched for known pfam models using HMMER (version 3.1b) (34). The pHMMs models were downloaded from Pfam (http://pfam.sanger.ac.uk 27.0) (35). They included Reverse transcriptase (*RVT_1*, PF00078; *RVT_2*, PF07727), Integrase core domain (*rve*, PF00665), Integrase DNA binding domain (*IN_DBD_C*, PF00552), Integrase Zinc binding domain (*Integrase_Zn*, PF02022), RNase H (*RNase_H*, PF00075), Retroviral aspartyl protease (*RVP*, PF00077; *RVP_2*, PF08284), and Retrotransposon gag protein (*Retrotrans_gag*, PF03732). If LTRs thus identified were flanked by TSDs and had internal coding domains sufficient to categorize the model to a superfamily, the LTR retrotransposon models were considered as full length elements. Based on the order of the RT and INT domains, individual elements were classified into *Copia* and *Gypsy* superfamilies. If both of these two domains could not be ascertained, individual elements were classified into LARDs (the large retrotransposon derivatives) and TRIMs (terminal repeat retrotransposons in miniature) superfamilies according to the length of the elements (LARD, > 4 kb; TRIM, < 4 kb) (36, 37).

### Definition of families of putative TEs

All putative TEs in the mulberry genome were classified into families based on the 80-80-80 rule. Two elements belonged to the same family if they shared at least 80% of sequence identity in at least 80% of their coding or internal domain, or within their terminal repeat region, or in both. Meanwhile, in order to prevent misclassification of short and possibly random stretches of homologous sequences, the shortest sequence should be longer than 80 bp (8).

### Annotation of putative TEs within the mulberry WGS assembly

RepeatMasker (http://www.repeatmasker.org, v 4.0.3) was used to find the distribution and coverage of the TEs in the mulberry WGS assembly. RMBlast was used as search algorithm with Smith-Waterman cutoff of 225. A custom Perl script (kindly provided by Robert Hubley, http://www.systemsbiology.org, Institute for Systems Biology) was used to automatically annotate the matched regions of the TEs in the genome by RepeatMasker in their respective TE superfamilies. The TEs abundance and coverage was calculated after filtering and annotation.

## Results

### Identification of TEs in mulberry

Using various methods and bioinformatics, we identified a total of 11 543 putative TEs in the mulberry genome, including 620 (PILER), 886 (RepeatModeler), 6156 (LTR_ FINDER), 1025 (LTR_STRUC), 890 (HelitroScanner), 37 (MGEScan-nonLTR), 198 (MITE-Hunter) and 1731 by similarity-based identification. Owing to the fact that TEs’ structures are complex and diverse; the identification of TEs in higher eukaryotic genomes is complicated and difficult. Further analyses of these mulberries putative TEs were carried out. To reduce the redundancy of similar prediction of PILER and RepeatModeler, we discarded putative TEs that have >90% sequence similarity to another prediction (signature-based identification and similarity-based identification). In addition, some of the putative TEs identified using above methods may have non-TE gene families, pseudogenes or highly repeated gene domains and needed to be filtered out. As a result, a total of 5925 TEs have been identified: 8 (PILER), 89 (RepeatModeler), 3545 (LTR_FINDER), 347 (LTR_STRUC), 33 (HelitroScanner), 36 (MGEScan-nonLTR), 136 (MITE-Hunter) and 1731 by similarity-based identification. Meanwhile, all these TEs were classified into 13 superfamilies, and 1062 families (Table 1).

**Table 1.** Summary of identified TEs in mulberry WGS assembly

Class	Order	Superfamily	Members	Families
Retrotransposons	LTR	*Copia*	1557	226
		*Gypsy*	1415	145
		*Lard*	722	312
		*Trim*	254	119
	LINE	*L1*	19	19
		*RTE*	30	30
DNA transposons	TIR	*PIF-Harbinger*	286	31
		*hAT*	1085	44
		*CMC*	249	38
		*MuLE*	136	39
		*TcMar*	1	1
	MITE	*MITE*	136	26
	Helitron	*Helitron*	35	32
Total			5925	1062

### Annotation of TEs in mulberry

In the mulberry genome, 143.17 MB (43.28 % of the assembly) sequences were annotated as TE-related sequences (Table 2). LTR retrotransposons of the superfamilies *Copia* (10.44%), *Gypsy* (9.20%) and *Lard* (8.59%) were the most abundant class of TEs, represented over 28% of the assembled mulberry genome. DNA transposons such as *MITE* (5.42%), *hAT* (2.88%), *CMC* (2.37%) and *PIF-Harbinger* (1.90%) were also identified. Prominent among these is the high proportion of *MITE*. We then compared the distribution of *MITEs* in mulberry to that in other sequenced Rosaceae species. As shown in Table 3, the *MITE* transposons occupied 5.42% of the mulberry assembly genome, which was comparable to that of apple (5.07%), pear (6.18%) and higher than that of strawberry (4.33%), and peach (3.89%). Recent genome wide duplications have shaped the genomes of apple (38) and pear (39). Such events have not undergone in the genomes of mulberry (13), strawberry (40) and peach (41). In this context, *MITE* elements were significantly enriched in the mulberry genome which lacks recent whole genome duplication. The expansion of this TE family during the evolution of mulberry would be candidates of interest for further study.

**Table 2.** Annotation of TE superfamilies in the mulberry WGS assembly

Class	Order	Superfamily	Masked (bp)	Percentage of Masked (%)	Percentage of genome (%)
Retrotransposons	LTR	*Copia*	34 541 580	24.13	10.44
		*Gypsy*	30 419 960	21.25	9.20
		*Lard*	28 414 859	19.85	8.59
		*Trim*	2 005 679	1.40	0.61
		unclassified	46 818	0.03	0.01
	LINE	*L1*	388 544	0.27	0.12
		*RTE*	974 028	0.68	0.29
	SINE	*tRNA*	680	0.00	0.00
DNA transposons	TIR	*PIF-Harbinger*	6 270 533	4.38	1.90
		*hAT*	9 525 810	6.65	2.88
		*CMC*	7 834 412	5.47	2.37
		*MuLE*	1 273 395	0.89	0.38
		*TcMar*	256 524	0.18	0.08
		*MITE*	17 917 995	12.52	5.42
		unclassified	42 381	0.03	0.01
	Helitron	*Helitron*	3 258 215	2.28	0.98
Total			143 171 413	100.00	43.28

**Table 3.** Comparison of the *MITE* in mulberry with other Rosaceae species

TEs		Mulberry	Apple	Pear	Strawberry	Peach
DTM	Element no	5378	158 680	33 701	13 789	16 178
	Total length (bp)	740 060	26 867 238	5 976 797	3 347 380	2 988 107
DTC	Element no		1874		140	
	Total length (bp)		496 676		21 797	
DTH	Element no	85 083	42 823	35 745	5197	12 371
	Total length (bp)	12 374 275	9 747 607	7 525 899	1 370 066	3 151 797
DTA	Element no	8532	32 324	17 297	7223	7315
	Total length (bp)	2 432 239	7 292 996	3 243 583	2 134 167	1 893 397
DTT	Element no	15 638		69 677		
	Total length (bp)	2 371 421		12 174 360		
DTx	Element no		1999	21563	8531	3246
	Total length (bp)		254 302	2525527	2 094 222	804 373
Total	Element no	114 631	237 700	177983	34 880	39 110
	Total length (bp)	17 917 995	44 658 819	31446166	8 967 632	8 837 674
	Genome size (MB)	330.79	881.28	508.55	206.89	227.25
	Percentage of genome (%)	5.42%	5.07%	6.18%	4.33%	3.89%

The *MITE* information of apple, strawberry and peach in this table were retrieved from plant MITE database (P-MITE, http://pmite.hzau.edu.cn/django/mite/) (47). The *MITE* information of pear was generated by using MITE-Hunter with default parameters. The consensus sequences generated by MITE-Hunter were manually checked using MSA files. Superfamilies are represented using different letters: DTT for *Tc1/Mar*, DTM for *MuLE*, DTA for *hAT*, DTC for *CMC*, DTH for *PIF-Harbinger* and DTx for unclassified superfamily.

### User interface

**Figure 1.**
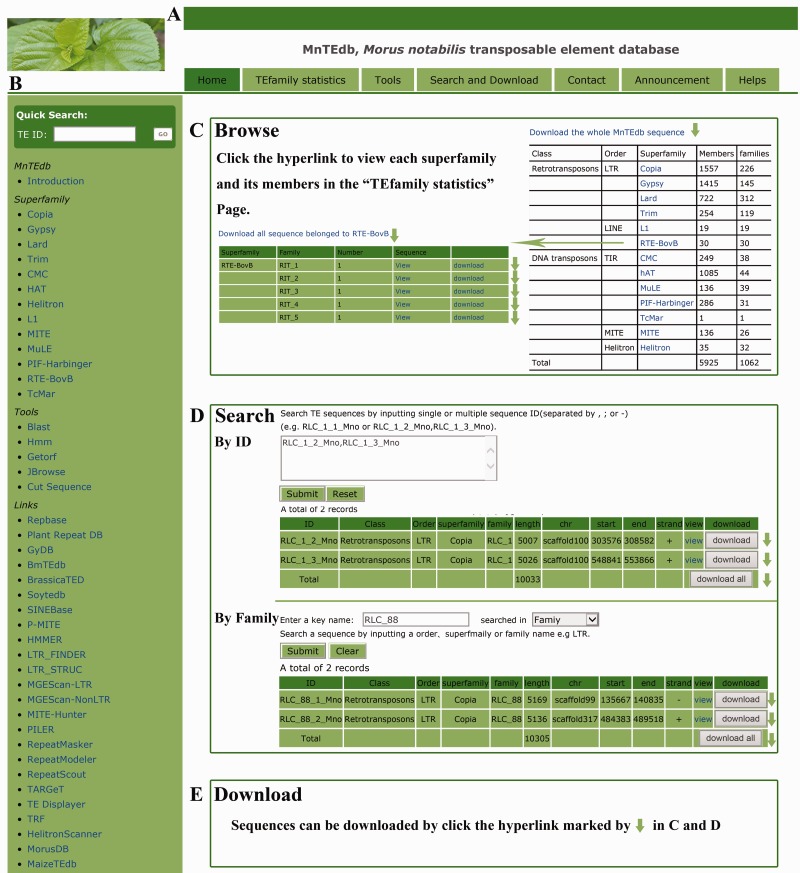
MnTEdb organization and the description of some functional sections in the database. **(A)** The top menu of MnTEdb. **(B)** The side menu of MnTEdb. **(C)** The user interface of browsing in MnTEdb. User can browse the detailed information of each superfamily and family by clicking the hyperlinks provided in this page. **(D)** The searching interface of MnTEdb. Two search approaches are provided for user in MnTEdb, including search using ‘ID’ and ‘Family’. All the search results can be shown under the search page. **(E)** Multiple approaches for TE sequences downloading have been provided. Data can be downloaded by clicking the marked region (green arrow).

In order to provide an efficient and user-friendly way to access the TE data, an easy-to-use web-based database, MnTEdb, was built to enable users to browse and search for the TE data and information, perform analyses using the analysis tools, and download all data of interest by clicking on hyperlinks on the page. The MnTEdb database organization is navigated by two menus: a top menu (Figure 1A) and a side menu (Figure 1B). The top menu contains four major sections: Browse, Search, Tools and Resources (Figure 1A). The side menu contains two major sections: Systematics and Links (Figure 1B).

### Browse

In the browsing interface, the basic information of the TEs in MnTEdb is shown. A total of 5925 full length TEs, which were grouped into different superfamilies, are shown on this page. Users can browse a superfamily of interest by the hyperlinks provided. The detailed information of each superfamily can be retrieved by clicking the corresponding entry (Figure 1C).

### Search

This section was developed to help users locate specific TEs in MnTEdb. Users can use a keyword (e.g. TE order, TE superfamily) to search the database. All the search results of interest to the users can be printed out as a tabular format output (Figure 1D). The search results can be downloaded by clicking the hyperlinks provided on the page.

### Tools

**Figure 2.**
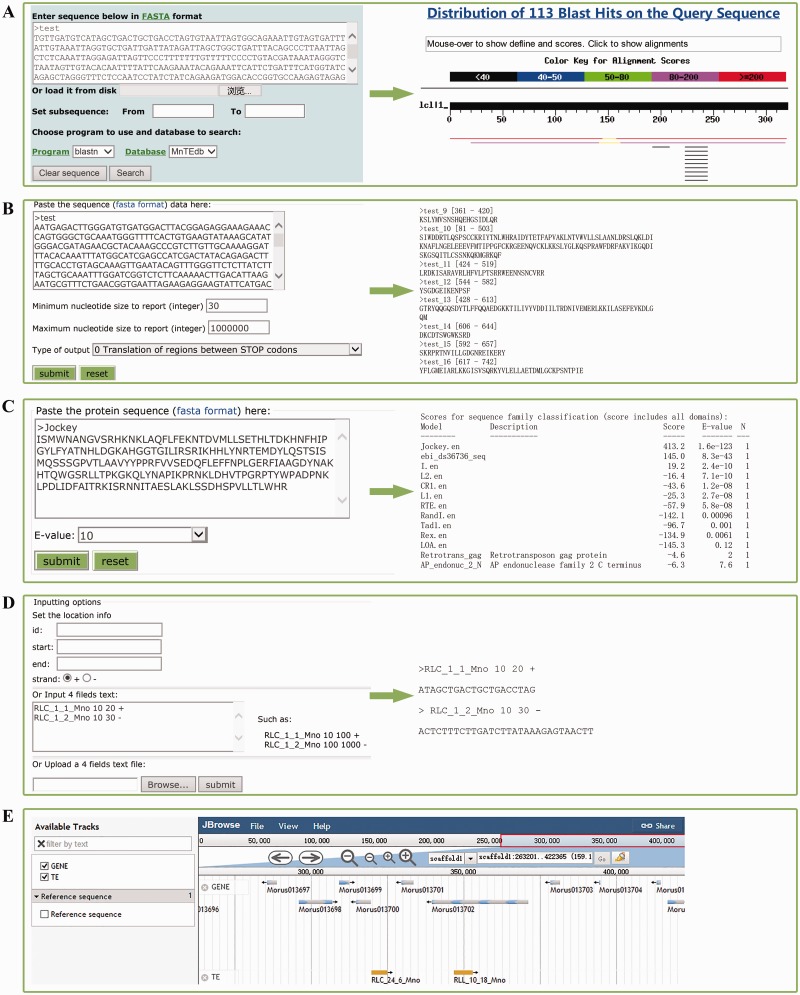
Snapshots of analysis tools provided in MnTEdb. **(A)** The BLAST interface (left) and a sample of BLASTn results (right). **(B)** The GetORF interface and the snapshots of the output results. **(C)** HMMER interface of a test protein sequence in MnTEdb. **(D)** An example of the input and output interface of the Sequence extractor. **(E)** Genome sequence view in JBrowse of a region in scaffold1. The gene models from the v1.0 genome version of *M. notabilis* were embedded in the Gene track.

Five types of tools, BLAST (Figure 2A) (42), GetORF (Figure 2B) [a subprogram from EMBOSS (33)], HMMER (Figure 2C) (43), Sequence extractor (Figure 2D) and JBrowser (Figure 2E) (44), were embedded in MnTEdb to help users mine, analyse and visualize the TE data. (i) BLAST. The standard wwwblast model was embedded. Users can submit the query sequences to perform a BLAST analysis against MnTEdb for a homology search (Figure 2A). (ii) GetORF. The potential ORF of the query sequences can be found by this program according to the parameters set by users (Figure 2B). (iii) HMMER. In this section, the HMM (Hidden Markov Model) profile of LTR and non-LTR retrotransposons coding domains were collected from previous studies (25, 45). The ORFs obtained by GetORF can be used as queries to search against these HMM files using HMMER package (http://hmmer.janelia.org, version 2.3.1), and to classify into corresponding superfamilies (Figure 2C). (iv) Sequence extractor. Users can fetch a sequence or sequences in a position defined by the users (Figure 2D). (v) JBrowse. We used the JBrowse genome browser tool to display the positional relationships between genes and TEs in the MnTEdb database. Two major levels are displayed: genes and TE information in the search area. Users can easily browse and search on a large scale in a graphic interface, and they can conveniently view and get detailed TEs as well as gene information (Figure 2E).

### Resources

In addition to the three sections described earlier (Browse, Search and Tools) multiple approaches for downloading of TE sequences were provided by MnTEdb. Users can download TE sequences by order, superfamily or family (Figure 1E).

### Systematics

In this section, users can browse and download detailed information of the superfamily in MnTEdb by clicking the corresponding hyperlinks. The information can be printed as a tabular format output (Figure 1B).

### Links

Finally, a variety of links to other database and software website initiatives relevant to MnTEdb were included in the side menu (Figure 1B).

## Discussion

Other database of *M.* *notabilis*, such as MorusDB (Morus Genome Database http://morus.swu.edu.cn/morusdb/), have mainly focused on genome data. MnTEdb was built to help users mining data from the TE sequences of mulberry easily and effectively. Compared with existing databases, it has its own specific features and advantages. (i) MnTEdb provided accurate and useful information for TEs in mulberry using multiple methods. It is an initial TE data repository for mulberry, other databases can use these data as basic data to develop their specific functions. (ii) MnTEdb provides features which are beneficial for analysis of TEs. For example, BLAST can be used for homology analysis of TEs, GetORF and HMMER can be used for the classification of TEs, and JBrowse can visualize the relation between TEs and genes. (iii) We encourage the submission of new TE data for mulberry. We will improve and continuously update the TE information, as well as research on TEs.

As more and more genome sequences become available, the number and types of TEs will grow. Therefore, MnTEdb will include TE data sets of all *Morus* species as they become available. Meanwhile, more and more evidence suggests that horizontal transfers of TEs are frequent and widespread in plants (46). MnTEdb will facilitate comparative analysis of TEs within the *Morus* genus to determine the role of TEs in the origin and evolution of *Morus* species.

## Conclusion

MnTEdb, a new and comprehensive database which focuses on the TE information of mulberry plant has been developed. Compared with other existing databases for mulberry, MnTEdb has its own specific features and advantages. It provides researchers with not only TE data but also tools for performing data analysis. In order to help users to fully and efficiently use the TE data of mulberry, we are committed to continuously improve its applications and embed more available TE data of *Morus* species in the future. MnTEdb will be a valuable resource for research into the comparative and evolutionary dynamics of TEs between *Morus* and other plants at the whole genome level.

### Availability

Database name: MnTEdb (http://morus.swu.edu.cn/mntedb/). All data deposited in the database are freely available to all users without any restrictions.
